# Genetic Variation and Its Reflection on Posttranslational Modifications in Frequency Clock and Mating Type a-1 Proteins in* Sordaria fimicola*

**DOI:** 10.1155/2017/1268623

**Published:** 2017-06-21

**Authors:** Rabia Arif, Faiza Akram, Tazeen Jamil, Hamid Mukhtar, Siu Fai Lee, Muhammad Saleem

**Affiliations:** ^1^Molecular Genetics Research Laboratory, Department of Botany, University of the Punjab, Lahore 54590, Pakistan; ^2^Institute of Industrial Biotechnology, Government College University, Lahore 54590, Pakistan; ^3^Department of Biological Sciences, Macquarie University and CSIRO Black Mountain Laboratories, Building 101, Clunies Ross Street, Black Mountain, ACT 2601, Australia

## Abstract

Posttranslational modifications (PTMs) occur in all essential proteins taking command of their functions. There are many domains inside proteins where modifications take place on side-chains of amino acids through various enzymes to generate different species of proteins. In this manuscript we have, for the first time, predicted posttranslational modifications of frequency clock and mating type a-1 proteins in* Sordaria fimicola* collected from different sites to see the effect of environment on proteins or various amino acids pickings and their ultimate impact on consensus sequences present in mating type proteins using bioinformatics tools. Furthermore, we have also measured and walked through genomic DNA of various* Sordaria* strains to determine genetic diversity by genotyping the short sequence repeats (SSRs) of wild strains of* S. fimicola* collected from contrasting environments of two opposing slopes (harsh and xeric south facing slope and mild north facing slope) of Evolution Canyon (EC), Israel. Based on the whole genome sequence of* S. macrospora*, we targeted 20 genomic regions in* S. fimicola* which contain short sequence repeats (SSRs). Our data revealed genetic variations in strains from south facing slope and these findings assist in the hypothesis that genetic variations caused by stressful environments lead to evolution.

## 1. Introduction

Environmental stress is thought to be among the key driving forces of evolution and species adaptation because of its influential role in inducing genetic variations which ultimately help the species to evolve by increasing their evolutionary potential [1–3]. Environmental conditions encounter organisms with natural selection by manipulating parental and genetic variants and thus genetic variations become a requirement for evolution as they determine the evolutionary potential of a population [4]. These variations in the form of base substitutions/mutations alter the expression of genes and ultimately generate more diverse frontier molecules such as proteins and glycoproteins through various posttranslational modifications which are fundamentally preferred by the organism facing the environmental stress. Developments in molecular biology have made it easier to explore genetic diversity of species by applying a number of potential tools in the form of molecular markers whereas simple sequence repeats (SSRs) are considered to be one of the strong candidates for detecting genetic diversity among species because they are largely interspersed in eukaryotic genomes, can easily be examined by PCR amplification with the help of unique flanking primers, and possess high levels of polymorphism [5, 6]. The SSRs act as codominant non-Mendelian markers that are more frequent and reproducible than dominant markers such as RAPD and are extensively used to determine genetic structure of population and genetic isolation of a population [7]. According to Borštnick and Pumpernik [8], SSR loci with tri- and hexamotifs are relatively abundant in coding regions of higher eukaryotic genomes and sequence polymorphism occurs in one or more repeat units of these loci due to insertion deletion mutations [9]. Little is known about SSRs in fungi with limited number of studies on these important and widespread sets of sequences. A survey of simple sequence repeats in sequenced fungal genomes by Karaoglu et al. [10] showed that the relative abundance of SSRs is low in fungi as compared to human genome and longer SSRs are also rare. For genetic characterization of* Aspergillus fumigatus* and* Saccharomyces cerevisiae* SSR markers have been considered superior by Pérez et al. [11] when compared with several other markers.

A vast range of posttranslational modifications cover functions of modified proteins leading to complexity of life. An increased knowledge about these potential PTMs is indispensable to make a better understanding of biological processes [12]. Different genes become active at different stages during cellular processes. Posttranslational modifications are involved in proper folding and maintenance of the 3D structures of protein [13]. It is hard to find out the 3D structures of protein experimentally due to constant variations in protein conformations as a result of intra- and intermolecular connections of proteins inside cell. Now we use computer based software to predict or to construct 3D structures. So, that ultimate impact of any environmental stress leading to altered gene expression through genetic variations can also be answered on the basis of changed 3D structures of proteins in order to understand the adaptive nature of a species in changing over patchy environment. In our study, we have predicted some major posttranslational modifications such as phosphorylation, glycosylation, and nuclear export signals sites on frequency clock and mating type a-1 protein of* S. fimicola *(saprophytic, coprophilous flask fungi). Frequency clock proteins are an important component of circadian clocks which control levels of gene expression that help to respond to different ecological conditions in many organisms and are particularly involved in rhythmic movements exhibited by many filamentous fungi. Mating type a-1 proteins, encoded by mat-a-1 genes, determine the sexual compatibility and vegetative incompatibility with A mating types in many ascomycetes. As understood many genes involved in sexual development of fungi are under the influence of* MAT* genes because these genes encode their transcriptional regulators [14]. Based upon the above-mentioned significance, mating type gene and frequency clock gene were targeted to calculate the posttranslational modifications. So far no PTMs have been calculated or predicted for mat proteins and PTMs for frequency clock proteins are experimentally known. As a result of this an attempt was made to predict PTMS in both proteins to compare the results with known findings.

In addition to this, we have also aimed to take advantage of the* S. macrospora* genome sequence and available bioinformatics tools to develop SSRs for* S. fimicola* and to investigate its genetic diversity in terms of environmental stress in Evolution Canyon, Israel, which bears contrasting environments in the form of two opposing slopes, that is, harsh and xeric south facing slope and mild north facing slope.

## 2. Materials and Methods

### 2.1. Organism

Stock cultures of all* S. fimicola* strains used in the present study were provided by Molecular Genetics Research Laboratory, Department of Botany, University of the Punjab, Lahore. These cultures were originally collected by Professor Nevo's colleagues [15] from south facing slope (SFS) and north facing slope (NFS) of Evolution Canyon, Israel. All the stock cultures were subcultured under sterile conditions and maintained on Potato Dextrose Agar (PDA) at 18°C (Supplementary Table 1 in Supplementary Material available online at https://doi.org/10.1155/2017/1268623).

### 2.2. Genomic DNA Extraction

Genomic DNA of all the strains was extracted by adopting high quality method of DNA extraction described by Pietro et al. [16] with some modifications (detailed steps of this method are provided in the supplementary data file), subjected to 1% agarose gel electrophoreses stained with ethidium bromide with ladder (Norgen 1 Kb ladder) DNA and photographed under gel documentation system (Ugenius3-SynGene). After extraction, g-DNA of all the strains was subjected to real time PCR for the amplification of indirect (SSR) and direct markers (frequency clock and mating type a-1 genes) to study genetic variations among the amplified products using Clustal Omega online alignment tool available at https://www.ebi.ac.uk/Tools/msa/clustalo/.

### 2.3. Real Time PCR

PCR amplifications were carried out for indirect (SSR) and direct markers (frequency clock and mating type a-1 genes) discretely using the Roche LightCycler® 480 systems. The 384-well plate was used for the PCR analyses. 10 *µ*l PCR mix is comprised of 2 *µ*l of genomic DNA (1 in 10 dilution of the g-DNA stock), 1 *µ*l of 10x PCR buffer (Bioline), 0.4 *µ*l of 50 mM MgCl_2_, 0.064 *µ*l of dNTPs, 0.25 *µ*l of the LightCycler 480 High Resolution Melting Master solution (Roche), 0.01 *µ*l of the IMMOLASE™ DNA polymerase (Bioline), 0.04 *µ*l of the forward and reverse primers each at 100 *µ*M, and 6.26 *µ*l of water.

The amplification was programmed as follows: initial DNA denaturing step of 95°C for 10 min, followed by 50 cycles of denaturation (95°C for 5 sec, 65°C for 15 sec, and 72°C for 1 min) ending with a final elongation step at 72°C for 5 min. Fluorescence acquisition was obtained after each 72°C step. Products were heated to 95°C for 1 minute, cooled to 40°C for one minute, and raised to 78°C for one second. As temperature increased gradually from 78°C to 95°C, fluorescence data were acquired continuously.

To amplify the frequency clock gene in different strains of* S. fimicola*, different pair of primers were designed to target the full length of targeted genes. NCBI's primer BLAST tool and Primer 3 were used to evaluate primer quality, specificity, and melting temperature. The details of primers used for amplification of frequency clock and mating type a-1 genes are given in Supplementary Table 2. WebSat, an online free program, was used to find SSRs from the survey of contig files obtained from whole genomic sequence of* S. macrospora* retrieved from https://www.ncbi.nlm.nih.gov/. Free WebSat tool is available at http://purl.oclc.org/NET/websat/. This web tool found SSRs up to tetramers. WebSat designed primers complementary to the flanking regions of microsatellites using Primer 3 program (Supplementary Figure 1). Twenty primer pairs were designed from the sequence data of the* S. macrospora* (Supplementary Table 3).

### 2.4. Posttranslational Modifications Prediction Tools

Posttranslational modifications were investigated with the help of various bioinformatics tools including YinOYang 1.2 Server (available at: http://www.cbs.dtu.dk/services/YinOYang/) NetPhos 3.1 Server (available at http://www.cbs.dtu.dk/services/NetPhos/), and NetNES 1.1 Server (available at: http://www.cbs.dtu.dk/services/NetNES/) to calculate YinOYang sites (the interplay between glycosylation and phosphorylation), phosphorylation sites, and nuclear export signals (NES), respectively. The amino acid sequences of amplified genes were obtained from online tool “EMBOSS Transeq” available at https://www.ebi.ac.uk/Tools/st/emboss_transeq/ while the amino acid sequences of all the reference strains, that is,* N. crassa, S. macrospora*, and* S. fimicola*, were retrieved from Uniprot available at: http://www.uniprot.org/proteomes/.

## 3. Results

**Figure 1:**
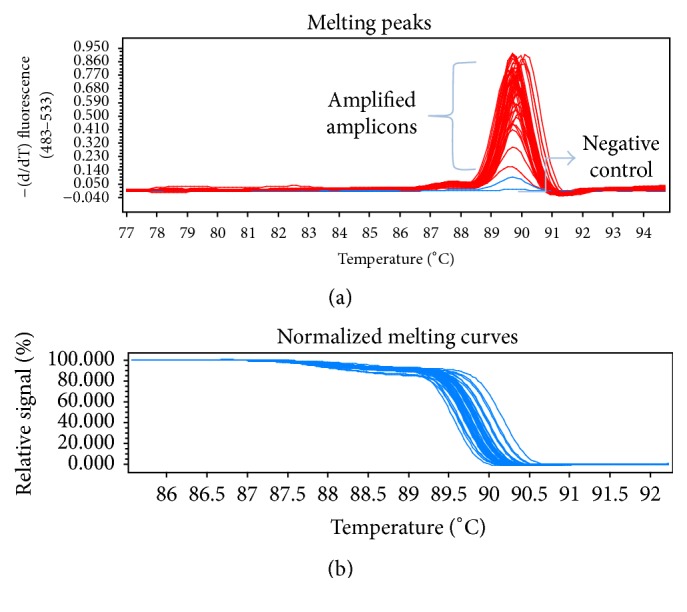
High Resolution Melt analysis for genotyping of frequency clock gene in fifty strains of* Sordaria fimicola *to calculate variations. (a) Melting peaks of fifty strains of* Sordaria fimicola *to calculate variations; most of the strains showed melting peaks at 90°C; few samples deviate from this range. A negative control was run to compare the amplifications. (b) Normalized melting curves of fifty strains of* Sordaria fimicola *to calculate variations.

**Figure 2:**
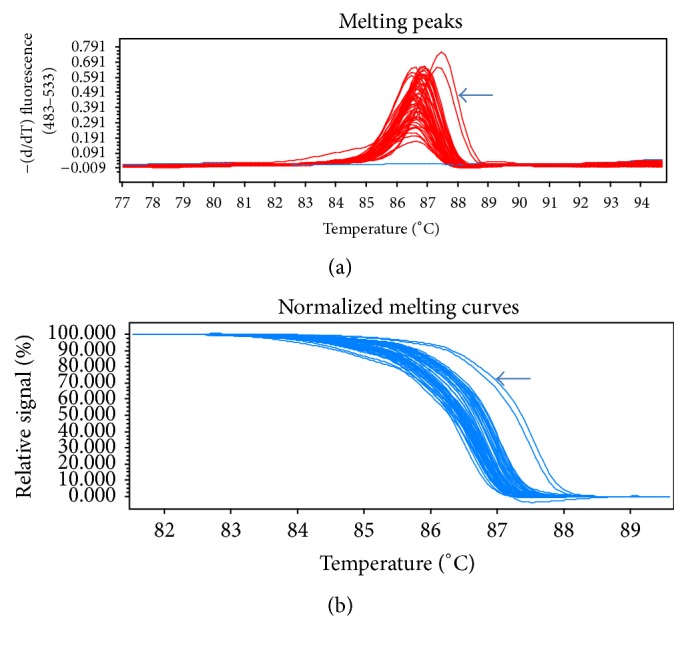
High Resolution Melt analysis for genotyping of mating type a-1 gene in fifty strains of* Sordaria fimicola *to calculate variations. (a) Melting peaks of fifty strains of* Sordaria fimicola *to calculate variations; most of the strains showed melting peaks at 87.5°C; few samples deviate from this range. (b) Normalized melting curves of fifty strains of* Sordaria fimicola *to calculate variations; arrow indicating samples that deviate from curve.

The genomic DNA of the parental strains of* S. fimicola* was subjected to the amplification of frequency clock and mating type a-1 genes and was analysed using high Resolution Melt analysis (Figure 1) for amplification by melting peaks and normalized melt curves (Figure 2). An amplified product of 1597 bp was obtained in strains isolated from north facing slopes while 1598 bp product was amplified in strains isolated from south facing slope for frequency clock gene. Point mutations on nucleotide 115 T(A), 947 G(T), and 948 T(G) position were observed in S2 and S3 strains, when aligned against reference sequence of* S. fimicola* using Clustal Omega (Table 1). Similarly, Mat-a-1 gene including exons 2 and 3 was amplified in all the strains and sequencing results were aligned. Three base substitutions were observed in mat-a-1 gene sequences and these changes ultimately altered the protein sequences of mat-a-1 genes. Base substitutions 234 T(A), 241 C(T), and 249 A(T) were observed in mat-a-1 gene sequences of south facing slope strains (Table 2).

All strains were also subjected to SSR-PCR to find out di-, tri-, or tetranucleotide potential short sequence repeats (SSRs). Based on HRM analysis, few PCR amplicons were run on 1.5% agarose gel to confirm the PCR amplifications. After sequencing, the sequences were subjected to BLAST tool at NCBI (https://www.Ncbi.nlm.nih.gov/BLAST) to check homologous sequences to those found for* S. fimicola*. Blastn searches were made. BLAST used* S. fimicola* sequence as query sequence to find out homologous region in* S*.* macrospora* (Table 3).

### 3.1. Prediction of O-Glycosylation and YinOYang Sites

O-glycosylation predicted sites for frequency clock protein are given in Table 4 attained by YinOYang 1.2. and YinOYang predicted sites are given in Table 5. These are the sites that are involved in interchange of phosphorylation and glycosylation.

### 3.2. Prediction of Phosphorylation and Nuclear Export Signals (NES)

**Figure 3:**
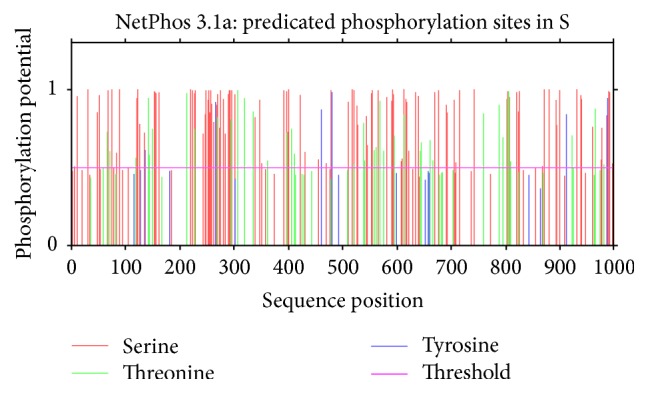
Predicted sites of phosphorylation on serine/threonine/tyrosine in* Sordaria fimicola for frequency clock protein.*

**Figure 4:**
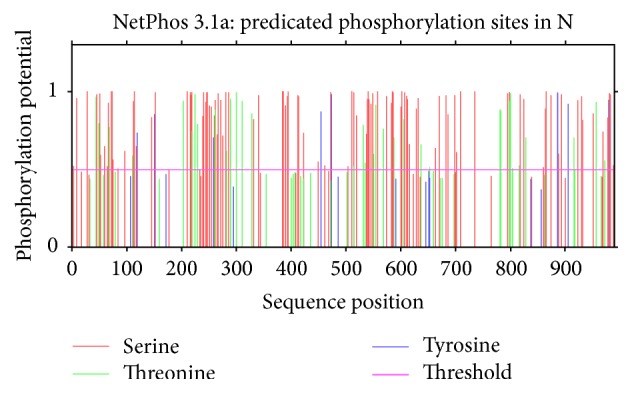
Predicted sites of phosphorylation on serine/threonine/tyrosine in* Neurospora crassa for frequency clock protein.*

**Figure 5:**
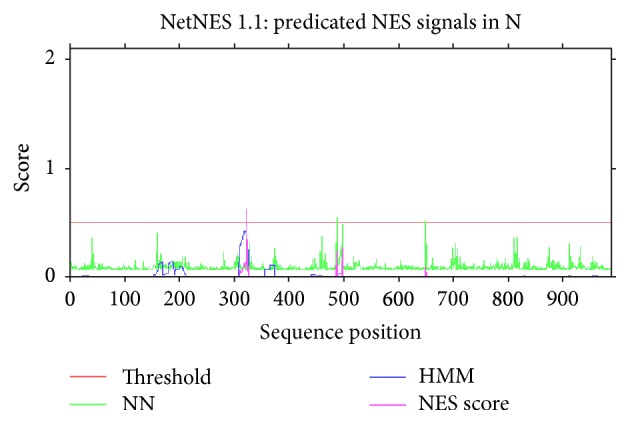
Predicted sites of leucine-rich nuclear export signals in* Neurospora crassa.*

**Figure 6:**
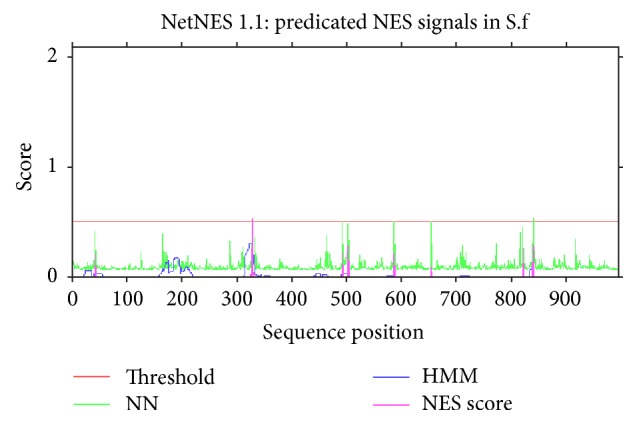
Predicted sites of leucine-rich nuclear export signals in* Sordaria fimicola.*

All possible phosphorylation sites (Figures 3 and 4) for frequency clock and mating type a-1 protein are given in Tables 6 and 7, respectively. Nuclear export signals on residue L-323 in* Neurospora* and residue L-328 in* S. fimicola* have been predicted for frequency clock protein (Figures 5 and 6) while the same sites in mating type proteins of all* Sordaria* strains are highly conserved.

## 4. Discussion

The potential of PTMs to change the configuration of proteins which affects their catalytic activities has been determined by many experiments through advanced techniques. There are many domains inside proteins where modifications take place on side-chains of amino acids through various enzymes to generate different classes of proteins. In order to cope with advancements in molecular biology, it has become crucial to understand how PTMs play an important role in maintaining biological functions of proteins [17]. This research on data refers to the possible posttranslational modifications on frequency clock and mating type a-1 proteins.

Phosphorylation, being one of the most significant posttranslational modifications, plays pivotal role in many biological processes including signal transduction pathways, metabolism, enzyme activities, cell proliferation, and apoptosis [12]. Our work has predicted 114 phosphorylation sites on serine, 35 on threonine, and 8 on tyrosine residue out of 989 amino acids of frequency clock protein of* N. crassa*, while 121 phosphorylation sites on serine, 41 on threonine, and 5 on tyrosine residues out of 998 amino acids of frequency clock protein in* S. fimicola* were predicted. By comparing the conserved region of frequency clock proteins with all other frequency homologs, it was found that phosphorylation on three amino acids, that is, T-501, S-513, and S-519, is conserved [18]. Matching conserved phosphorylation on the same above-mentioned amino acids is predicted in* N. crassa* using NetPhos 3.1 software (Figure 3) in present investigation. Phosphorylation of similar pattern is also predicted in* S. fimicola* but at different positions, that is, T-506, S-518, and S-524 (Figure 4). Phosphorylation on these three positions is experimentally known by many workers. Modifications on T-501 and S-519 do not show any substantial effect on deprivation of short frequency clock (SFRQ) protein as compared to modification on amino acid S-513. Phosphorylation on serine-513 has dramatic effect on circadian clock constancy and degradation. Taken together, these data strongly suggest that phosphorylation triggers the degradation of FRQ, and that the degradation rate is a determining factor for the period length of the circadian clock. Despite the existence of additional phosphorylation sites, Ser-513 appears to be one of the main sites for determining the degradation rate of FRQ [19] which is Ser-518 in case of* S. fimicola.* Phosphorylation of FRQ proteins occurs immediately after their synthesis and continues until the protein is confronted by some degradation pathway [19, 20]. For instance, S-8, S-28, S-50, T-139, and T-304 are predicted as potential phosphorylation sites of* S. fimicola* in Table 6. These sites have a score of 0.9 indicating a very likely phosphorylation site, whereas Y-596 has a score of only 0.4 below the threshold (0.500) and indicates the fact that the confidence for this site being a true phosphorylation site is quite low. Interestingly, phosphorylation appears to play a similar role for a* Drosophila* clock protein, PER, which is also progressively phosphorylated over time. DBT, a casein kinase I homolog, leads either directly or indirectly to the phosphorylation and degradation of PER, because PER is hypophosphorylated [21]. Frequency clock proteins being a component of circadian rhythms when phosphorylated can have impact on sclerotia formation in* Aspergillus flavus* and enzyme rhythms in* A. nidulans* [22]. Yang et al. [23] have also reported phosphorylation of this protein in* N. crassa*. Baker et al. [24] and Tang et al. [25] have reported more than 85 individual phosphorylation sites in same protein of* N. crassa*. So, the predicted modifications in* S. fimicola* frequency clock protein may have same implications as described in* N. crassa* and other ascomycetes. Liu [26] has reported that phosphorylation of frequency clock proteins occurs immediately after its synthesis by several kinases and two types of phosphatases, PP1 and PP2A, dephosphorylate it.

Like phosphorylation, glycosylation, O-GlcNAc change, is an active and a controlling method that can prevent phosphorylation on the similar serine and or threonine residues which may be obligatory for regulation of many biological processes. These modifications lead to the formulation of Yin Yang assumptions [27], which implies that such alterations or modifications fight for the similar position on a polypeptide. According to our results, frequency clock protein of* N. crassa* has 27 sites (Supplementary Figure 2) with the potential of interplay between O-glycosylation and phosphorylation while* S. fimicola *(Supplementary Figure 3) has 25 such sites (Tables 4 and 5). Multiple sequence alignment of frequency clock proteins of* S. fimicola* and* N. crassa* is given in Supplementary Figure 4.

Nuclear export signals are very crucial components of biological molecules as they regulate their subcellular localization. Majority of the export from nucleus to cytoplasm depends upon these signals such as various transcription factors and proteins. Easily accessible and flexible attributes of these signals help other factors and proteins to interact with them in order to move out to the cytoplasm [28]. Prediction of these signals at position 328 of frequency clock protein in reference sequence of* S. fimicola* and position 323 in the same protein from reference strain of* N. crassa* is an indication of regulation of this protein through nuclear export signals (Figures 5 and 6). No nuclear export signal was found in any strain of* S. fimicola*.

Mating type genes regulate many sexual and asexual events in the life cycle of fungi [29]. They impart mating type properties to the particular fungal strains including sexual development, compatibility and incompatibility with other strains, and secretion of pheromones as they encode many transcriptional regulators which mediate the expression of genes involved in sexual development [14]. Among the idiomorphs of mating type genes, mating type a-1 encodes the MT a-1 polypeptide which helps in binding and regulating specific DNA sequences because it encloses an HMG box domain (a DNA binding motif found in high mobility group proteins and a diverse set of regulatory proteins) and any mutation within this domain results in loss of mating ability [30]. Another segment of mating type a-1 gene encodes the perithecium maturation function [31]. We have predicted phosphorylation of mating type a-1 proteins in the similar way as for frequency clock protein using NetPhos 2.0 software. The differences in partially amplified mating type a-1 sequences of N5 and S3 strains of* S. fimicola* ultimately generated different amino acid sequences of mating type a-1 protein after translation and these sequences are not only altered at amino acid level but also at phosphorylation sites. S3 strain and reference strain of* S. fimicola* showed phosphorylation at 3 serine residues and 2 tyrosine residues while N5 strain was found to be phosphorylated at 4 serine residues and 2 tyrosine residues as shown in Table 7. Nuclear export signals are highly conserved among all the tested strains of* Sordaria* as they are predicted on similar residues in all strains. Since mating type proteins from any other source have not yet been worked out for posttranslational modifications experimentally, so PTMs predicted in our work appear not to be backed up to suggest some genuine implications of these modifications/functions of mating type a-1 proteins.

We have also performed SSRs analysis for wild strains of* S. fimicola* collected from contrasting environments (i.e., south facing slope: SFS and north facing slope: NFS) of Evolution Canyon in order to explore the impact of environment on genetic diversity and our results revealed that the number of SSR motif repeats varies among some strains from these contrasting environments. Out of the 20 primers tested, 11 primers amplified SSRs in targeted DNA of various* Sordaria* strains. The sequence analysis showed that although* S. fimicola* amplicons showed high homology to their corresponding regions in* S. macrospora* and contained the expected SSR motifs, the primer pair 336257193 (F+R) amplified a sequence of S3 with one more SSR motif, that is, (GTG)_6_, as compared to N5 with (GTG)_5_ motif repeat. Other than this variation, no remarkable differences were obtained among other strains. Such types of SSR-linked polymorphisms have been reported in other eukaryotes [32] and were proven to be effective in detecting genetic diversity [33]. Faria [34] carried out characterization of a novel set of 20 microsatellite markers with the help of* Eucalyptus *EST databases. These markers were found to be transferable and polymorphic through 6 major* Eucalyptus* species. Microsatellites (SSRs) and RAPD markers have been used by Nevo [35] to explore genetic diversity between wild strains of wheat* (Triticum dicoccoides)*. Shahida et al. [36] have also assessed genetic diversity among wild strains of* S. fimicola* using random amplified polymorphic DNA (RAPD) technique and found that the strains from south facing slope were more diverse and polymorphic as compared to north facing slope. Hosid et al. [37] have reported high levels of polymorphism in ascomycete's soil fungus* Emericella nidulans *from stressful and arid environment with the help of SSR markers. Thorough literature study does suggest that this is the first attempt to explore the genetic diversity of* S. fimicola* in natural populations of Evolution Canyon using SSR markers. Arif et al. [38] targeted 16 regions from* S. macrospora* containing short sequence repeats to genotype these SSRs flanking region in different natural strains of* S. fimicola* and found 12 homologous regions similar to that of* S. macrospora*. They found high enrichment of SSRs motif and variation in motifs number in strains isolated from south facing slope of EC as compared to strains from north facing slope of EC. Genetic diversity of* S. fimicola* from Evolution Canyon has already been reported by Lamb et al. [1] and Saleem et al. [2] on the basis of gene conversions and frequency of spontaneous mutations with a conclusion that wild strains from south facing slope prove to be harbouring more mutations because they exhibit higher frequency of crossing over and spontaneous mutations. In addition to SSR variations, we have also identified variations in the nucleotide sequences of frequency clock and mating type a-1 genes amplified in* Sordaria* strains from SFS and NFS (Tables 1 and 2). Although our findings reveal relatively low genetic variations which are not good enough to correlate with environmental stress, however these findings suggest south facing slope (SFS) to be a bit more mutagenic than north facing slope that might be due to higher rates of radiations and extreme temperature as explained by Lamb et al. [1] and Saleem et al. [2].

In the current research, frequency clock gene sequences from* S. fimicola* were submitted to NCBI database under accession numbers KY026774, KY026775, KY026776, and KY000835 for* S. fimicola* strains N5, S2, S3, and N6, respectively.

## Supplementary Material

Supplementary Table 1: List of Parental Strains and their F_1_ generation used for evaluation of potential SSR markers for S. *fimicola* collected from two contrasting slopes SFS and NFS of EC.
Supplementary Table 2: List of primers for the amplification of Frequency Clock and mating type 1 genes in *Sordaria fimicola*.
Supplementary Table 3: List of SSR primers used for *Sordaria fimicola*.
Supplementary Figure 1: WebSat output for finding SSRs and for the design of primer pair 266426263-F and 266426263-R to amplify the (CT)_7_ microsatellite locus.
Supplementary Figure 2: Prediction of O-beta-Glycosylation in Neurospora crassa.
Supplementary Figure 3: Prediction of O-beta-Glycosylation in Sordaria fimicola.
Supplementary Figure 4: Multiple sequence Alignment of frequency clock protein between Sordaria fimicola and Neurospora crassa.

## Figures and Tables

**Table 1:** Multiple Sequence alignment of four parental strains with reference sequence of *Sordaria fimicola* under the accession number L14467 to observe nucleotide variations. Underlined area indicates point mutation between nucleotides of frequency clock gene.

S.f	TTGTGAACGAATTTAGCGCACCCCTGCGAAATATGACGTTAGATGTGGGACATTT $\underline{T}$ GTCA	120
N6	TTGTGAACGAATTTAGCGCACCCCTGCGAAATATGACGTTAGATGTGGGACATTT $\underline{T}$ GTCA	120
N5	TTGTGAACGAATTTAGCGCACCCCTGCGAAATATGACGTTAGATGTGGGACATTT $\underline{T}$ GTCA	120
S2	TTGTGAACGAATTTAGCGCACCCCTGCGAAATATGACGTTAGATGTGGGACATTT $\underline{A}$ GTCA	120
S3	TTGTGAACGAATTTAGCGCACCCCTGCGAAATATGACGTTAGATGTGGGACATTT $\underline{A}$ GTCA	120
	∗ ∗∗∗∗∗∗∗∗∗∗∗∗∗∗∗∗∗∗∗∗∗∗∗∗∗∗∗∗∗∗∗∗∗∗∗∗∗∗∗∗∗∗∗∗∗∗∗∗∗∗∗∗∗∗ ∗∗∗∗	
S.f	GGGGCAGAAAAATCAGTCAATTGAAAGATAGGAACAAGACCGGCTGCC $\underline{GT}$ ATCCTGGACG	960
N6	GGGGCAGAAAAATCAGTCAATTGAAAGATAGGAACAAGACCGGCTGCC $\underline{GT}$ ATCCTGGACG	958
N5	GGGGCAGAAAAATCAGTCAATTGAAAGATAGGAACAAGACCGGCTGCC $\underline{GT}$ ATCCTGGACG	958
S2	GGGGCAGAAAAATCAGTCAATTGAAAGATAGGAACAAGACCGGCTGCC $\underline{TG}$ ATCCTGGACG	959
S3	GGGGCAGAAAAATCAGTCAATTGAAAGATAGGAACAAGACCGGCTGCC $\underline{TG}$ ATCCTGGACG	959
	∗∗∗∗∗∗∗∗∗∗∗∗∗∗∗∗∗∗∗∗∗∗∗∗∗∗∗∗∗∗∗∗∗∗∗∗∗∗∗∗∗∗∗∗∗∗∗∗ ∗∗∗∗∗∗∗∗∗∗	

**Table 2:** Multiple sequence alignment of parental strains of *Sordaria fimicola* to observe nucleotide variations for mating type A1 gene. Underlined area indicates point mutation between nucleotides. *∗* indicates conserved region.

N5	ATTTCAGCATGGCCAATGAGATCAAGGCTAGATTGTTGCTGGACAATCCCGAC $\underline{T}$ TCGCTA	240
N6	ATTTCAGCATGGCCAATGAGATCAAGGCTAGATTGTTGCTGGACAATCCCGAC $\underline{T}$ TCGCTA	240
N7	ATTTCAGCATGGCCAATGAGATCAAGGCTAGATTGTTGCTGGACAATCCCGAC $\underline{T}$ TCGCTA	240
Sw92.1	ATTTCAGCATGGCCAATGAGATCAAGGCTAGATTGTTGCTGGACAATCCCGAC $\underline{T}$ TCGCTA	240
S1	ATTTCAGCATGGCCAATGAGATCAAGGCTAGATTGTTGCTGGACAATCCCGAC $\underline{A}$ TCGCTA	240
S2	ATTTCAGCATGGCCAATGAGATCAAGGCTAGATTGTTGCTGGACAATCCCGAC $\underline{A}$ TCGCTA	240
S3	ATTTCAGCATGGCCAATGAGATCAAGGCTAGATTGTTGCTGGACAATCCCGAC $\underline{A}$ TCGCTA	240
Sw17.1	ATTTCAGCATGGCCAATGAGATCAAGGCTAGATTGTTGCTGGACAATCCCGAC $\underline{A}$ TCGCTA	240
	∗∗∗∗∗∗∗∗∗∗∗∗∗∗∗∗∗∗∗∗∗∗∗∗∗∗∗∗∗∗∗∗∗∗∗∗∗∗∗∗∗∗∗∗∗∗∗∗∗∗∗∗∗ ∗∗∗∗∗∗	

N5	$\underline{C}$ AATCTAG $\underline{A}$ GATGATGCTAGTCTTGGCCCA-	266
N6	$\underline{C}$ AATCTAG $\underline{A}$ GATGATGCTAGTCTTGGCCCAC	266
N7	$\underline{C}$ AATCTAG $\underline{A}$ GATGATGCTAGTCTTGGCCCAC	266
Sw92.1	$\underline{C}$ AATCTAG $\underline{A}$ GATGATGCTAGTCTTGGCCCA-	266
S1	$\underline{T}$ AATCTAG $\underline{T}$ GATGATGCTAGTCTTGGCCCAC	266
S2	$\underline{T}$ AATCTAG $\underline{T}$ GATGATGCTAGTCTTGGCCCAC	266
S3	$\underline{T}$ AATCTAG $\underline{T}$ GATGATGCTAGTCTTGGCCCAC	266
Sw17.1	$\underline{T}$ AATCTAG $\underline{T}$ GATGATGCTAGTCTTGGCCCAC	266
	∗∗∗∗∗∗∗ ∗∗∗∗∗∗∗∗∗∗∗∗∗∗∗∗∗∗∗∗∗∗	

**Table 3:** Multiple sequence alignment of eight parental strains of *Sordaria fimicola* with reference sequence of *Sordaria macrospora* for genotyping of SSRs (GTG)7 by primer 336257193 (F/R).

S2	AATACCGTTTCCGACGCTAGTAGTAGTGGTGGTGGTGGTGGTGGTGGTCGGCTTGGTCGG
S3	AATACCGTTTCCGACGCTAGTAGTAGTGGTGGTGGTGGTGGTGGTGGTCGGCTTGGTCGG
Ref	AATACCGTTTCCGACGCTAGTAGT $\underline{G}$ GTGGTGGTGGTGGTGGTGGTGGTCGGCTTGGTCGG
S1	AATACCGTTTCCGACGCTAGTAGTAGTGGTGGTGGTGGTGGTGGTGGTCGGCTTGGTCGG
N5	AATACCGTTTCCGACGCTAGTAGTAGTGGTGGTGGTGGTG-------TCGGCTT------
N6	AATACCGTTTCCGACGCTAGTAGTAGTGGTGGTGGTGGTG------GTCGGCTTGGTCGG
N7	AATACCGTTTCCGACGCTAGTAGTAGTGGTGGTGGTGGTG------GTCGGCTTGGTCGG
Sw92.1	AATACCGTTTCCGACGCTAGTAGTAGTGGTGGTGGTGGTGGTGGTGGTCGGCT-------
Sw17.2	AATACCGTTTCCGACGCTAGTAGTAGTGGTGGTGGTGGTGGTGGTGGTCGGCTTGG----
	∗∗∗∗∗∗∗∗∗∗∗∗∗∗∗∗∗∗∗∗∗∗∗∗ ∗∗∗∗∗∗∗∗∗∗∗∗∗∗∗ ∗∗∗∗∗∗

**Table 4:** Prediction results of O-glycosylation sites of frequency clock protein in *Sordaria fimicola* and *Neurospora crassa.*

Server	Organism	Predicted glycosylation sites	
		On serine	On threonine
YinOYang 1.2	*Sordaria fimicola*	4, 28, 45, 46, 51, 65, 66, 82, 151, 152, 222, 240, 246, 250, 251, 252, 261, 267, 271, 288, 289, 356, 395, 515, 518, 822, 830, 866, 930, 973, 985, 995, 997	57, 64, 78, 92, 139, 268, 292, 427, 506, 867, 964
	*Neurospora crassa*	4, 28, 45, 51, 59,65, 66, 72, 145, 146, 216, 234, 240, 244, 245, 246, 248, 255, 261, 265, 271, 272, 510, 513, 519, 541, 582, 662, 817, 824, 860, 977, 987, 988, 989	44, 57, 262, 282, 422, 501, 534, 537, 551, 781, 816, 861, 956
	SFS	4, 46, 51, 65, 66, 82, 151, 152, 222, 240, 246, 250, 251, 252, 261, 267, 271, 277, 278, 288, 289, 356, 395, 515, 822, 830, 866, 930, 985, 995, 996, 997	57, 64, 139, 147,268, 292, 427, 786, 821, 867, 923, 964, 973
	NFS	4, 26, 45, 46, 51, 65, 66, 82, 151, 153, 222, 240, 246, 250, 251, 252, 262, 267, 271, 288, 356, 395, 515, 518, 822, 830, 866, 930, 973, 985,	57, 64, 139, 268, 292, 427, 506, 786, 964

**Table 5:** Prediction results of YinOYang sites of frequency clock protein in *Sordaria fimicola* and *Neurospora crassa.*

Server	Organism	Predicted YinOYang sites	
		On serine	On threonine
YinOYang 1.2	*Sordaria fimicola*	28, 45, 66, 151, 152, 222, 240, 246, 250, 251, 252, 261, 267, 268, 271, 277, 288, 289, 395, 515, 822 Total: 21	64, 139, 786, 964, 985 Total: 5
	*Neurospora crassa*	28, 45, 66, 72, 145, 216, 234, 240, 244, 245, 245, 246, 248, 255, 261, 265, 271, 510, 537, 541, 582, 662 Total = 22	44, 262, 956 Total: 3
	SFS	28, 45, 66, 151, 152, 222, 240, 246, 247, 250, 252, 261, 267, 271, 277, 289, 395, 515, 822, 985 Total: 20	64, 139, 268, 786, 964 Total: 5
	NFS	28, 45, 151, 152, 240, 246, 247, 250, 251, 261, 267, 271, 277, 289, 395, 515 Total: 16	64, 139, 268, 786 Total: 4

**Table 6:** Prediction results of phosphorylation sites of frequency clock protein in *Sordaria fimicola* and *Neurospora crassa*.

Server	Organism	Predicted phosphorylation sites		
		On serine	On threonine	On tyrosine
NetPhos 3.1	*Sordaria fimicola*	4, 8, 28, 45, 46, 50, 66, 72, 73, 76, 82, 86, 119, 120, 124, 133, 151, 152, 153, 159, 217, 221, 222, 224, 225, 240, 44, 246, 250, 251, 252, 253, 254, 256, 261, 265, 267, 271, 272, 277, 278, 281, 286, 288, 289, 291, 293, 294, 299, 337, 346, 349, 389, 390, 395, 397, 399, 419, 428, 454, 459, 467, 477, 478, 508, 515, 518, 524, 525, 542, 543, 550, 553, 556, 559, 563, 579, 587, 589, 591, 606, 607, 611, 615, 617, 626, 637, 667, 674, 688, 690, 703, 706, 713, 740, 800, 802, 805, 818, 822, 823, 830, 866, 869, 870, 879, 891, 898, 910, 929, 936, 937, 939, 958, 974, 976, 985, 988, 989, 990, 996 Total: 121	64, 68, 117, 139, 141, 147, 210, 225, 268, 292, 304, 316, 333, 359, 394, 404, 409, 506, 519, 536, 539, 566, 574, 593, 610, 641, 642, 658, 664, 757, 786, 787, 794, 803, 804, 808, 821, 910, 923, 964, 979 Total: 41	134, 263, 842, 911, 987 Total: 5
	*Neurospora crassa*	4, 8,28, 45, 50, 51, 52, 59, 65, 66, 68, 72, 73, 76, 85, 97, 113, 114, 118, 145, 153, 211, 215, 216, 218, 220, 234, 238, 240, 244, 245, 246, 247, 248, 250, 255, 259, 261, 265, 266, 271, 275, 280, 286, 332, 341, 384, 385, 390, 392, 399, 412, 414, 423, 449, 462, 472, 503, 510, 513, 519, 537, 538, 540, 541, 545, 548, 558, 573, 574, 582, 584, 586, 600, 601, 602, 606, 610, 612, 615, 627, 632, 662, 669, 683, 685, 698, 701, 708, 734, 735, 795, 797, 800, 817, 818, 824, 866, 863, 864, 873, 885, 887, 892, 904, 923, 929, 931, 950, 968, 977, 980, 981, 988 Total: 114	44, 49, 111, 204, 219, 225, 230, 262, 282, 289, 299, 311, 328, 501, 514, 531, 534, 551, 554, 569, 588, 605, 636, 637, 653, 781, 782, 798, 794, 803, 816, 828, 915, 956, 971 Total: 35	120, 151, 257, 454, 473, 886, 905, 979 Total: 8

**Table 7:** Prediction of posttranslational modifications in different strains and reference sequence of *Sordaria fimicola* for mating type a-1 protein.

Server used to predict PTMs	Serial number	Mat-A				S.F
		S2	S3	N5	N6	
Glycosylation						
YinOYang	1	—	—	—	—	101S^*∗*^
Phosphorylation						
NetPhos3.1	1	41Y	41Y	39Y	41Y	68Y
	2	43S	43S	41S	43S	70S
	3	59Y	59Y	57Y	59Y	86Y
	4	66S	66S	64S	66S	93S
	5	74S	74S	72S	74S	101S
				74S	76S	
				75S	80T	
Nuclear export signals						
NETNES 1.1	1	48I	48I	48I	48I	75I
	2	49K	49K	49K	49K	76K
	3	50A	50A	50A	50A	77A
	4	51R	51R	51R	51R	78R
	5	52L	52L	52L	52L	79L
	6	53L	53L	53L	53L	80L
	7	54L	54L	54L	54L	81L

*∗* refers to YinOYang sites where the phosphorylation and glycosylation interplay.
