# Supplementary material for: Genetic Variation and Its Reflection on Posttranslational Modifications in Frequency Clock and Mating Type a-1 Proteins in Sordaria fimicola
Source: Biomed Res Int. 2017 Jun 21;2017:1268623. doi: 10.1155/2017/1268623 (PMC5499255; doi:10.1155/2017/1268623)
Supplement: Supplementary file 1 — Supplementary Table 1: List of Parental Strains and their F1 generation used for evaluation of potential SSR markers for S. fimicola collected from two contrasting slopes SFS and NFS of EC. Supplementary Table 2: List of primers for the amplification of Frequency Clock and mating type 1 genes in Sordaria fimicola. Supplementary Table 3: List of SSR primers used for Sordaria fimicola. Supplementary Figure 1: WebSat output for finding SSRs and for the design of primer pair 266426263-F and 266426263-R to amplify the (CT)7 microsatellite locus. Supplementary Figure 2: Prediction of O-beta-Glycosylation in Neurospora crassa. Supplementary Figure 3: Prediction of O-beta-Glycosylation in Sordaria fimicola. Supplementary Figure 4: Multiple sequence Alignment of frequency clock protein between Sordaria fimicola and Neurospora crassa. [file 1268623.f1.docx]

**Supplementry Material**

**Supplementary Table 1:** List of Parental Strains and their F_1_ generation used for evaluation of potential SSR markers for S*. fimicola* collected from two contrasting slopes SFS and NFS of EC

| Sr. no. | S2 | S3 | N6 | N5 | S_W_ 92.1 |
| --- | --- | --- | --- | --- | --- |
|  | RS_2_ 34.6 | RS_3_ 04.2 | RN_6_ 17.2 | RN_5_ 06.5 | RS_W_ 80.1 |
|  | RS_2_ 69.1 | RS_3_ 42.3 | RN_6_ 10.5 | RN_5_ 10.7 | RS_W_ 76.2 |
|  | RS_2_ 22.2 | RS_3_ 06.3 | RN_6_ 30.1 | RN_5_ 15.5 | RS_W_ 66.5 |
|  | RS_2_ 11.3 | RS_3_ 14.5 | RN_6_ 54.5 | RN_5_ 27.4 | RS_W_ 60.7 |
|  | RS_2_ 46.4 | RS_3_ 27.6 | RN_6_ 56.8 | RN_5_ 42.3 | RS_W_ 90.3 |
|  | RS_2_ 40.1 | RS_3_ 09.2 | RN_6_ 61.3 | RN_5_ 19.6 | RS_W_ 92.5 |
|  | RS_2_ 56.8 | RS_3_ 15.7 | RN_6_ 70.7 | RN_5_ 07.8 | RS_W_ 43.8 |
|  | RS_2_ 86.7 | RS_3_ 21.8 | RN_6_ 80.4 | RN_5_ 20.2 | RS_W_ 50.5 |
|  | RS_2_ 71.5 | RS_3_ 31.5 | RN_6_ 33.7 | RN_5_ 04.1 | RS_W_ 87.1 |
|  | RS_2_ 11.6 | RS_3_ 36.1 | RN_6_ 46.2 | RN_5_ 05.5 | RS_W_ 89.3 |

RS, RN, RSw, indicating progeny raised by Rabia from a single spore of original Strains of S_2,_ S_3,_ N_5_ N_6_ S3, S_W_ 92.1. RS_2_ (F_1_ generation of S_2_), RS_3_ (F_1_ generation of S_3_), RN_5_ (F_1_ generation of N_5_), RN_6_ (F_1_ generation of N_6_) and RS_W_ (F_1_ generation of S_W_ 92.1). First number indicated octade number, full stop and second number referred position of spore in an ascus. For initial screening only eight parental strains (S1, S2, S3, N5, N6, N7, Sw17.2 and Sw92.1) were used. Sw stands for Sordaria white mutants.

**Supplementary Table 2:** List of primers for the amplification of Frequency Clock and mating type 1 genes in *Sordaria fimicola*

| Freq_F1 | AGCTCAAGATACTTTGGGTCG |
| --- | --- |
| Freq_R1 | CCCGACACGACTCGATAAATTTGA |
| Freq_F2 | ATAGGAACAAGACCGGCTGC |
| Freq_R2 | TGAGACGGATGAGGTTGTGC |
| mat_a1_F | CAGAAGAAGAAGGCCAAGATTCCTC |
| mat-a1-R | ATTGTAGCGATAGTCGGGATTGTCC |

**Supplementary Table 3:** List of SSR primers used for *Sordaria fimicola*

| Primer | Forward | Reverse | SSR | Repeat | Expected product (bp) | SSR found |
| --- | --- | --- | --- | --- | --- | --- |
| 266426263 | ACTTGCGGTACCTGTATTCCCAT | ATGAGACCAATGTCGATGAATG | CT | 7 | 249 | Yes |
| 240247748 | CCGTCTGAATGAGTTTCTGTTG | CATAGAGGTAGGTTTTCACCGCT | TCC | 6 | 353 | Yes |
| **226885242** | AGTGGGATTTACACGTTCTGCT | CCCTGATGCTGTTCCTATTGAT | AC | 7 | 351 | Yes |
| **226815604** | CGTCAATCAGAAATCTTCGCGTC | GGCCCATCTTTCAAGTCTCA | CCA | 6 | 350 | Yes |
| **146327844** | CCCTTCCTCTTTTCTCTCCATT | TTGACCTTTCTCTTCCTTGCTC | CAT | 8 | 398 | Yes |
| **193227756** | ATTCAAGCTGCATCATCATCAC | CTCTATGTAGTATGCTCCCCGC | CAT | 8 | 337 | Yes |
| **164510147** | TTAGTCAAGAGAGACCGGACCAT | GAGTTGGAGTTGAAGTTGAGGG | CGA | 6 | 363 | Yes |
| **164510145** | CAACATTGAGCCAAAGGAAGAT | CTCAGGGGTGAAGAAGGTAATG | CAT | 12 | 373 | No |
| **110566586** | AGAATCCAGAAGAGACGAGACG | AAGTGGAAAGTGGCTGAAAGAG | TCCC | 6 | 387 | No |
| **8218219** | AACACAACACAAAACAAACGAGC | CTGCCATCAGAGTACACACACA | GAG | 4 | 300 | No |
| **39645990** | CTACAACCTT CCCAAGAACC CT | GAAGAGGGAGCCGGAGAGGC | CAA | 18 | 363 | Yes |
| 336257193 | TCTGGATAGGGGTAGGCGTCTG | GGCTCAACCTGCAATGGACTCT | GTG | 7 | 362 | Yes |
| 336241528 | CCATTCACATCATTCGTGACCC | CGTTTACGCTATGGGCAACACA | TGG | 7 | 350 | Yes |
| 336241251 | AACGACCACGAACGAATCCAAT | GCGCCGTGACAAGTTCTATCAA | AT | 11 | 386 | No |
| 336239778 | ACGACAGCACAACCACTCTTCG | AGATGGAGGAGGAGGAGGAGGA | GAA | 7 | 345 | No |
| 336257403 | ACCCAAGAGGGAGGAAAAGACG | CGTTTACGCTATGGGCAACACA | TTG` | 11 | 363 | Yes |
| 380086584 | AATAGGCTTGGTTCCCGCCTTA | TCGTTCTGACGGAGGCATTAAAA | TA | 6 | 360 | No |
| 380086579 | CGACTAACAGTGGGTGCCAAGA | ATAAAGAGCGAACCGTGAACGC | AT | 8 | 342 | NO |
| 380086569 | GTACCTATGCCCAGCCGCC | AGCTGTCCCAGTTAGGCAGATCA | TTCA | 6 | 387 | NO |
| 380085080 | ACTCCTCCTCCTCCTGCCGA | GCGATCTCCTCTGGACTAAAATTGC | TAA | 6 | 340 | No |


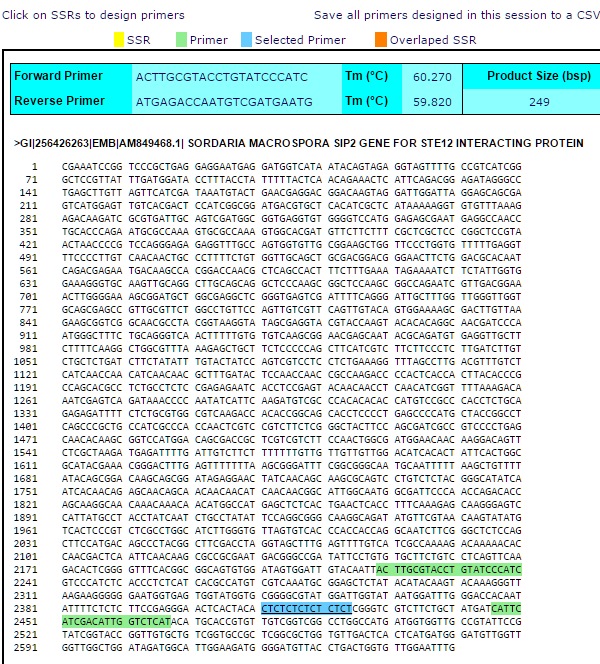


**Supplementary Figure 1**: WebSat output for finding SSRs and for the design of primer pair 266426263-F and 266426263-R to amplify the (CT)_7_ microsatellite locus.


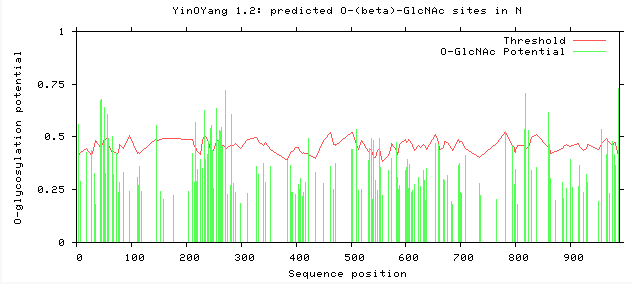


**Supplementary Figure 2: Prediction of O-beta-Glycosylation in Neurospora crassa**


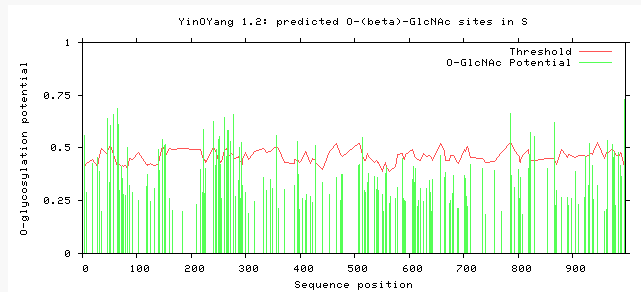


**Supplementary Figure 3: Prediction of O-beta-Glycosylation in Sordaria fimicola**

N MADSGDKSQGMRPPPFDSRGHPLPRRASPDKSITLENHRLARDTSSRVTSSSALGVTESQ 60

S MADSGDKSQGMRPPPFDSRGHPLPRRASPDKSITLHNHRLARDASSRVISSSALGVTGPQ 60

***********************************.*******:**** ******** *

N PQLKSSPTRRNSSG------ESEPTNWFNQSNRNPAAAFHDESHIMEVDPPFYQKETDSS 114

S PQPTSSPTRRDSSGESNDTGQSDPKSWFDQSNRNPVAAFNDESNIMEVDPPFYQKETDSS 120

** .******:*** :*:*..**:******.***:***:****************

N NEESRYPPGRNPVHPPGGVQLPGFRPVAAHSSAADDYRSVIDDLTVENKRLKEELKRYKQ 174

S NEDSRYPPGRNPSYPPRDTQTQGFRATVAHSSSADDFRSVIDDLTVENKRLKEELKRYKQ 180

**:********* :** ..* *** ..****:***:***********************

N FGSDVMRKEKLFEIKVHGLPRRKKRELEATLRDFAASLGDSSESTSQRRKTGRHGTAVHS 234

S FGSDMMRKEKLFEIKVHGLPRRKKRELEATLREFAASLGDSSESTSQRRKAGRHGKAVHS 240

****:***************************:*****************:****.****

N SGVSLSKHDSSSSSRSRPVDSAYNSMSTGRSSHAPHSSGPSLGRPSLTRAKSVGTQKVEN 294

S SGVSLSKHDSSSSSRSRPVDSAYASMSTGRSSHAAHSSGPSLGRPSLSS-KSTSSQKVES 299

*********************** ********** ************: **..:****.

N YLRDTPDGLLPHHIVMTDKEKKKLVVRRLEQLFTGKISGRNMQRNQSMPSMDAPLAPEGT 354

S YLRDTPDGLLPHHVVMTDKEKKKLVVRRLEQLFTGKISGRNMQRIPSIPSMDAALVSEGT 359

*************:****************************** *:***** *. ***

N NMAPPRPPPEGLREACIQLQDGDNPRKNRSSKDNGSASNSGGDQTELGGTGTGSGDGSGS 414

S IMAPPRPPPEGSREACIQLQEGENPEKIRSSKGATSASNSGGDQTEVGGTVTAGGDGNGS 419

********** ********:*:**.* ****. ***********:*** *..***.**

N GGRTGNNTSPPGAIAPDQRPTRPRDLDPDRVQIPSENMDYIRHLGLVSPEFLQGSRTSYQ 474

S GGRTVNNTSPPGVIAPDQRPTRPRDLDPDRVQIPSENMDYIRHLGLVSPEFLQGSRTSYQ 479

**** *******.***********************************************

N DVAPDAEGWVYLNLLCNLAQLHMVNVTPSFIRQAVSEKSTKFQLSADGRKIRWRGGTDGT 534

S DVAPDAEGWVYLNLLCNLAQLHMINVTPSFIRQAVSEKSTKFQLSSDGRKIRWRGGTDGT 539

***********************:*********************:**************

N KFSSDSSEDKSQQSPMTEDTEDGSDKNGRRKKRKTQQASSEIGRFGPSRSPSDTFHYKPM 594

S KFSSDNGEDKSQKSPLTDDTEDGSDKTGRRKKQKTQQARSEIGRLGLSRSPSDTFHYKPM 599

*****..*****:**:*:********.*****:***** *****:* *************

N FVHRNSSSIETSLEESMSQGSEDAVDESNMGNSKWDFSGSGTTQQRRKRRYDGAIVYYTG 654

S FVHCHASSAETSLEESASQGSEDFVDESNLANSKWDFSGSGTTQQRRKRRYDGAIVYYTG 659

*** ::** ******* ****** *****:.*****************************

N APFCTDLSGDPGDMSPTAQMTAGREVEGSGSGDEVEHVLQRTLSGSSLPIRPLSDDRARV 714

S AQFCTDLSGEPGDMSPTEQMTATGEQEASGSGDEAGRVLQRTLSGSSLLVRPLSDDRARV 719

* *******:******* **** * *.******. :*********** :**********

N AEVLDFDPGNPPELVADDGSSPNDEDFVFPWCEDPAKVRIQPIAKEVMEPSGLGGVLPDD 774

S AEALDFDPQNPPDLVSDDGFSPNDEDFVFPWCEDPAKTQVQPLAKEVMGRSGLGGVLPDD 779

**.***** ***:**:*** *****************.::**:***** **********

N HFVMLVTTRRVVRPILQRQLSRSTTSEDTAEFIAERLAAIRTSSPLPP-RSHRLTVAPLQ 833

S HFAIFVTTRRVMRPTLQRHLSRSTTSEDTAEIIAERLASIRTSSPLPPPRSRNLILAPLQ 839

**.::******:** ***:************:******:********* **:.* :****

N VEYVSGQFRRLNPAPLPPPAIFYPPFSTDSSWDDGDDLASDDEEVEEVEEDSYSEGQISR 893

S IEYVAGEFHRLNPASLPPPAMFYPPFSTDSSWDDGDDLVSVEEEVEEMEEESFSEGQMSR 899

:***:*:*:***** *****:*****************.* :*****:**:*:****:**

N RANPHFSDNNTYMRKDDLAFDTETDVRMDSDDN--RLSDSGHNMRAMMPRAEAVDGDDSP 951

S RANPHFSDNNTYMRKEDLAFDTETDVRMDSSHDHRMSSDSGLMMRSVMRRPVAVDGDGSP 959

***************:**************..: **** **::* * *****.**

N LAAVTGKEVDMLHTGSSVATAGGAESGYSSSMEDVSSS 989

S LATVTGRDVDMLHTSSSVATAGGAESGYSSSMEDVSSS 997

**Supplementary Figure 4: Multiple sequence Alignment of frequency clock protein between Sordaria fimicola and Neurospora crassa**

**Supplementary Data file**

**Genomic DNA extraction**

Genomic DNA isolation of *S. fimicola* (for all generations and strains) was done by Modified Pietro method (Pietro *et al*., 1995).

**Material/chemicals Required**

Eppendorf tubes (1.5 ml); Micropipettes, Pestle and Mortar, Vortex, Water bath, Centrifuge machine, Incubator, Liquid nitrogen, Spirit, Choloroform: Isoamyl alcohol (25:24:1), 70% v/v ethanol, Isopropanol, Lysis buffer, Sterile ultrapure water, 2% CTAB buffer, TE buffer

**Lysis buffer**

Lysis buffer is prepared by mixing following 3 buffers:

**BUFFER A**

0.35M sorbitol, 0.1M Tris-HCl pH 9, 5mM EDTA 2.5 vols, of A (5ml)

**BUFFER B**

0.2 M Tris-HCl, pH 9, 2 M NaCl, 2% CTAB, 50 mM EDTA, pH 8, 2.5 vols, of B (2ml)

**BUFFER C**

5% Sarkosyl, 1 vols, of C (2ml)

**Proteinase K**

Buffer A, B and C were mixed and Proteinase K was added to this.

**Procedure**

1. Mycelia (about 10-15mg) from mature petriplates were scraped with the help of sterile needle or spatula and transferred in an eppendorf and let them to freeze at

-20^0^C for overnight.

1. After overnight soaking, sample was transferred to pestle and mortar and crushed by adding liquid nitrogen.
2. 300-500µl of Lysis buffer containing 0.1mg proteinase K was added to crush material to grind the mycelium thoroughly. Crushed material was transferred to eppendorf and vortexed briefly.
3. Eppendorf containing crushed sample was incubated for 1 hour at 65^0^C in a water bath.
4. Centrifuged the eppendorf at 13000g for 15 min. After centrifugation ~600µl of supernatant was shifted to a new eppendorf tube with the help of a micropipette.
5. Proteins were removed from supernatant by sequential extraction using 600µl Phenol: Chloroform: Isoamyl alcohol in a ratio of 25:24:1 v/v. Centrifuged the eppendorf at 13000g for 15 min and then the upper aqueous layer was transferred to a new eppendorf.
6. Aqueous layer was centrifuged at 13000g/ 15 min. by adding Chloroform: Isoamyl alcohol in a ratio of 24:1 v/v.
7. The aqueous supernatant layer ~600 µl was shifted to a new eppendorf; 1 ml of isopropanol was added, mixed thoroughly with the help of vortexer. Eppendorf was incubated at -70^0^ C for 1 hour or overnight at -20^0^ C
8. After incubation eppendorf was centrifuged at 13000g/15 min. Supernatant was drained with micropipette while the pellet was retained.
9. DNA pellet was washed with 150µl of 70% v/v ethanol for two times. Discarded the supernatant layer of ethanol and the pellet was allowed to dry at room temperature.
10. DNA pellet was dissolved in 30-50µl of ultra-sterile, double distilled pure water or in Tris EDTA buffer.
11. DNA pellet was subjected to PCR procedure immediately. Or the solution e.g., DNA was stored at -20^0^ C., if not to use immediately.
